# The Inside Story of Adenosine

**DOI:** 10.3390/ijms19030784

**Published:** 2018-03-09

**Authors:** Marcella Camici, Mercedes Garcia-Gil, Maria Grazia Tozzi

**Affiliations:** 1 Dipartimento di Biologia, Unità di Biochimica, Via San Zeno 51, 56127 Pisa, Italy; maria.grazia.tozzi@unipi.it; 2 Dipartimento di Biologia, Unità di Fisiologia Generale, Via San Zeno 31, 56127 Pisa, Italy; mercedes.garcia@unipi.it

**Keywords:** adenosine, adenosine kinase, adenosine deaminase, adenosine receptors, energy repletion, deoxyadenosine, transmethylation

## Abstract

Several physiological functions of adenosine (Ado) appear to be mediated by four G protein-coupled Ado receptors. Ado is produced extracellularly from the catabolism of the excreted ATP, or intracellularly from AMP, and then released through its transporter. High level of intracellular Ado occurs only at low energy charge, as an intermediate of ATP breakdown, leading to hypoxanthine production. AMP, the direct precursor of Ado, is now considered as an important stress signal inside cell triggering metabolic regulation through activation of a specific AMP-dependent protein kinase. Intracellular Ado produced from AMP by allosterically regulated nucleotidases can be regarded as a stress signal as well. To study the receptor-independent effects of Ado, several experimental approaches have been proposed, such as inhibition or silencing of key enzymes of Ado metabolism, knockdown of Ado receptors in animals, the use of antagonists, or cell treatment with deoxyadenosine, which is substrate of the enzymes acting on Ado, but is unable to interact with Ado receptors. In this way, it was demonstrated that, among other functions, intracellular Ado modulates angiogenesis by regulating promoter methylation, induces hypothermia, promotes apoptosis in sympathetic neurons, and, in the case of oxygen and glucose deprivation, exerts a cytoprotective effect by replenishing the ATP pool.

## 1. Introduction

Intracellular free nucleotides originate mainly in the liver through de novo synthesis pathways [1]. As for many other compounds, liver synthesizes nucleotides for exportation, and, since de novo synthesis produces phosphorylated compounds, they must be dephosphorylated into nucleosides and in part phosphorolytically cleaved into bases and ribose-1-phosphate (Rib-1-P), in order to leave the hepatocyte, enter the blood flux, and be taken up by cells and organs in the body. Therefore, adenosine (Ado) is synthesized mainly in the liver as the product of dephosphorylation of AMP, and travels in the blood at a concentration of about 0.5 µM [2,3]. Eventually, Ado enters cells mainly through equilibrative nucleoside transporters (ENT) and is phosphorylated by Ado kinase (AdoK) and adenylate kinases into ADP. The newly synthesized ADP and ADP, arising from the kinase reactions in which ATP is used as a phosphate donor, then enter mitochondria for oxidative phosphorylation [3]. Therefore, the uptake of Ado requires an active oxidative metabolism to be driven.

**Figure 1:**
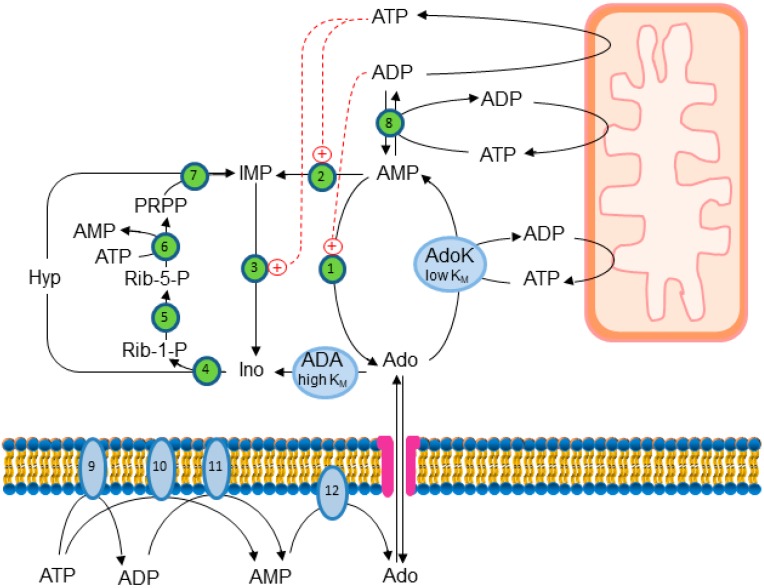
Interplay between internal and external Ado production. Ado originates intracellularly from AMP predominantly in case of low energy charge, and can be exported or deaminated. Extracellular Ado stemming from ATP breakdown may enter the cell and is readily phosphorylated by the low K_M_ adenosine kinase (AdoK). The high K_M_ adenosine deaminase (ADA) comes into play if Ado accumulates. 1: cytosolic 5′-nucleotidase I (cN-I); 2: AMP deaminase; 3: cytosolic 5′-nucleotidase II (cN-II); 4: purine nucleoside phosphorylase (PNP); 5: phosphoribomutase; 6: PRPP synthetase; 7: hypoxanthine guanine phosphoribosyltransferase (HPRT); 8: adenylate kinase; 9,11: ecto-nucleoside triphosphate diphosphohydrolase; 9: ecto-nucleotide pyrophosphatase/phosphodiesterase; 12: ecto-5′-nucleotidase.

Extracellular Ado can also arise from intracellular ATP degradation. In fact, as shown in Figure 1, at high energy charges, ATP degradation mainly produces inosine (Ino) and hypoxanthine (Hyp) since, in the presence of high ATP concentration, AMP is mainly deaminated into IMP, which is dephosphorylated into Ino. In fact, both AMP deaminase and the cytosolic 5′-nucleotidase II (cN-II) are allosterically activated by ATP [4,5]. On the contrary, at low energy charge, AMP can accumulate inside the cell at high micromolar concentrations, thus activating a specific AMP-dependent protein kinase (AMPK), the master regulator of cellular energy homeostasis [6]. It can also be dephosphorylated by a high K_M_ AMP specific 5′-nucleotidase I (cN-I), which is strongly activated by ADP, leading to Ado accumulation inside the cell [7]. In these conditions, Ado can undergo deamination, but the K_M_ of Ado deaminase (ADA) for Ado is high enough (25–150 µM) [8,9] to allow for Ado accumulation and exportation through ENTs [10]. Therefore, Ado can be generated inside the cell and exported in the external medium, in the same conditions in which AMP is accumulating, opening up a possibility for the nucleoside to act as a danger signal, both interacting with specific receptors on the same cell or on the surrounding cells and acting intracellularly. Finally, intracellular Ado concentration can increase following the catabolism of extracellular ATP; indeed, the extracellular Ado stemming from ATP rapidly equilibrates with the intracellular compartment (Figure 1). As a result of the regulation of Ado metabolism, the nucleoside cannot accumulate in healthy cells at very high concentrations without being readily deaminated into Ino. Ino, in turn is cleaved into Hyp and Rib-1-P by purine nucleoside phosphorylase (PNP). Hyp can be salvaged as IMP or excreted as uric acid, while the phosphorylated sugar can be utilized for 5-phosphoribosyl-1-pyrophosphate (PRPP) synthesis, energy repletion, or glucose synthesis (see Section 5). As has long been known, dietary purines are dephosphorylated in the intestinal lumen, and the resulting nucleosides are actively transported inside intestinal mucosa and catabolized, leading to uric acid and Rib-1-P production. In fact, intestinal mucosa expresses a high amount of purine catabolic enzymes such as ADA, PNP, and xanthine oxidase [11]. As mentioned before, Rib-1-P may be utilized for energy repletion, while the purine ring is converted into uric acid. On the other hand, when one of the enzymes involved in Ado catabolism and particularly ADA is defective, a substantial amount of Ado and deoxyadenosine (dAdo), both of dietary and intracellular origin, is circulating in the blood and may enter cells and organs. Deficiency of ADA is the most common cause of severe combined immunodeficiency (SCID) caused by a systemic accumulation of Ado and dAdo, which are cytotoxic particularly for immune systems (see Section 4). It is therefore important to maintain the homeostatic concentration of Ado. In fact, its accumulation can be dangerous since critical cellular functions or processes may be dysregulated. Indeed, Ado plays several physiological functions through receptor-dependent or -independent mechanisms. Cell-surface Ado receptors are coupled to the G-protein family [12]. These receptors were initially classified on the basis of their pharmacologic response profiles to agonism by adenosine analogs and antagonism by methylxanthines as determined by their ability to inhibit (A1 and A3 subtypes) or stimulate (A2 subtype) adenylate cyclase [13]. The A2 adenosine receptor (A2AR) subtype was subsequently further categorized according to the presence of high-affinity (A2_A_AR) or low-affinity (A2_B_AR) binding sites for Ado in the brain [14]. To date, four Ado receptor subtypes have been identified, purified, cloned, and expressed from mouse, rat, human, and other mammalian as well as non-mammalian species [15]. The reader is referred to the numerous excellent reviews covering the different aspects of Ado receptor expression, function, and regulation [16,17]. This article focuses on the intracellular metabolism of Ado, which maintains the homeostatic concentration of the nucleoside, and on the receptor-independent effects of Ado, summarizing the reports obtained using several experimental approaches, such as inhibition or silencing of key enzymes of Ado metabolism, knockdown of Ado receptors in animals, the use of antagonists, or cell treatment with dAdo, which is a substrate of the enzymes acting on Ado but is unable to interact with Ado receptors.

## 2. Hypothermia Induced by Adenylate Compounds

Ado is known to cause profound hypothermia mainly through interaction with A1ARs coupled to an inhibitory G-protein [18,19]. Although the transduction pathway of A1AR is well known, how the activation of this pathway induces hypothermia is largely unknown. Both A1AR and A3AR have been reported to cause hypothermia in mice [20]. AMP can also induce hypothermia, possibly after its conversion to Ado or directly through interaction with A1AR in human cells and mice [21,22]. Zhang et al. [23] suggest that in mice the hypothermia resulting from an increase in extracellular AMP may be induced by a parallel change in the intracellular nucleotide concentration, which may allosterically regulate relevant metabolic enzymes, such as AMPK, fructose 1,6 bisphosphatase, and phosphofructokinase. Indeed, ecto-5′-nucleotidase dephosphorylates extracellular AMP to Ado, which enters the cell by ENT and is readily phosphorylated to AMP by the low K_M_ AdoK (Figure 1). However, since Ado is an intermediate, it is very difficult to distinguish whether its effect is receptor-mediated or consequent to its internalization and possible metabolic conversion to other intermediates. Of course, if an effect exerted by Ado persists in animals knockout for specific Ado receptors, we may conclude that those effects are mediated by a receptor-independent way, thus presumably by a mechanism directly or indirectly depending on intracellular Ado metabolism. Studies utilizing the A1AR^−/−^, A3AR^−/−^, and double knockout mice indicate that selective A1AR agonists induce hypothermia through both A1AR and A3AR. In addition, fasting-induced torpor in mice does not require any of these receptors, but the mechanism remains unknown [20]. Eisner et al. [24] demonstrate that treating mice with Ado or AdoK inhibitors (thus obtaining an increase of Ado) causes a profound hypothermia lasting longer if caused by AdoK inhibitors. The same experiment performed in A1AR^−/−^ mice caused a less pronounced effect that is 40–60% of that observed in wild type mice. Experiments with selective agonists indicate that the activation of A2AR and A3AR contributes only marginally to hypothermia. Furthermore, ADA inhibitors are unable to induce hypothermia and do not affect the hypothermic effect of AdoK inhibitors on both wild type and A1AR^−/−^ mice, thus indicating that the deamination of Ado is not required for the nucleoside to induce hypothermia. The authors conclude that hypothermia induced by Ado in mice is a combined response to a receptor-mediated (mainly A1-mediated) mechanism and to a still-unknown intracellular mechanism in which AdoK plays a fundamental regulatory role [24].

## 3. Intracellular Ado in the Central Nervous System

Ado is a neuromodulator that fine-tunes brain neurotransmission mainly acting through inhibitory A1ARs and facilitatory A2_A_ARs [15,25]. A1ARs are abundantly expressed throughout the brain, controlling synaptic transmission [17]. A1AR activation depends on the Ado concentration in the tissue [26], and AdoK activity is a key regulator of endogenous Ado activating A1ARs [27]. In accordance with their inhibitory role curtailing excitatory transmission, bolstering A1AR activation through inhibition of AdoK affords neuroprotection against brain damage involving glutamate excitotoxicity, epilepsy, and brain ischemia [27].

In the murine adult brain, removal of Ado occurs largely in astrocytes by AdoK [27,28,29]. The extracellular concentration of Ado is determined by neuronal Ado release and AdoK-driven reuptake through ENTs in astrocytes, which form a “sink” for Ado [30]. Disruption of Ado homeostasis and Ado deficiency have been implicated in epileptogenesis [31,32], and local increase of Ado has been demonstrated to be an effective strategy to acutely suppress seizures in animal models of epilepsy [27,30].

**Figure 2:**
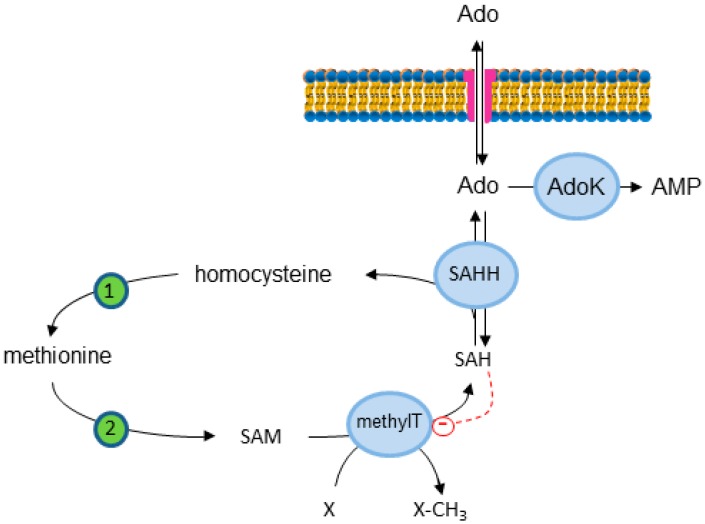
Transmethylation pathway. *S*-Adenosylhomocysteine (SAH) is hydrolyzed to homocysteine and Ado by *S*-adenosylhomocysteine hydrolase (SAHH). 1: Methionine synthase. 2: Methionine adenosyl transferase catalyzes the formation of *S*-adenosylmethionine (SAM). SAM is the donor of methyl group in the transmethylation reactions catalyzed by methyltransferases (MethylT). X: acceptor (protein, DNA, RNA) of the methyl group. SAH is an inhibitor of MethylT (dashed line). Ado can exit or enter the cell through the ENT.

Intracellular Ado can be removed by ADA or by AdoK (Figure 1). However, as assessed in Section 1, because of its much lower K_M_ [33], AdoK is regarded as the principal enzyme regulating intracellular Ado concentrations under physiological conditions [27]. AdoK dysfunction has been reported to be involved in several pathologies including epilepsy and cancer. With the aim to investigate the role of Ado in the brain, several transgenic mice with AdoK knockout or overexpressing AdoK through the entire brain or in specific regions have been obtained. Dysregulation of brain Ado in transgenic mice with brain-wide or telencephalon AdoK overexpression resulted in working memory deficiency and impaired Pavlovian conditioned freezing [34], while mice with brain-wide deletion of AdoK develop spontaneous seizures and profound deficits in hippocampus-dependent learning and memory [35]. Many actions of Ado are mediated by Ado receptors and are therefore blocked by using antagonists of the receptors. For example, blocking A2_A_AR activity can attenuate neurological symptoms in the brain-wide AdoK knockout transgenic mice [35]. However, there is evidence that intracellular glial Ado plays a role in many neuronal receptor-mediated and receptor-independent effects. Some of these functions depend on the effect on the transmethylation pathway illustrated in Figure 2. As an example, we will describe the influence of glial Ado in modification of neuronal DNA methylation in epilepsy and in sleep homeostasis.

### 3.1. Transmethylation Pathway

Elevated Ado regulates DNA methylation through interference with the transmethylation pathway. If metabolic clearance of Ado through AdoK is impaired, *S*-adenosylhomocysteine (SAH) levels rise. SAH in turn is known to inhibit DNA methyltransferases (DNMT) through product inhibition [36] (Figure 2). Ado is an anticonvulsant and acts through presynaptic and postsynaptic A1ARs to decrease neuronal excitability [37]. In the kainic acid, murine model of temporal lobe epilepsy, disruption of Ado homeostasis (increased AdoK and reduced Ado) is accompanied with an increase in hippocampal DNA methylation, which correlates with increased DNA methyltransferase activity, and spontaneous recurrent seizures [38]. On the other hand, chronic administration of the AdoK inhibitor 5-iodotubercidin leads to a decrease in global DNA methylation in the mouse hippocampus. This hypomethylation is maintained in mice with a genetic disruption of the A1AR, suggesting that the hypomethylation is not an A1AR-dependent effect. Similarly, the transgenic mice (fb-Adk-def mice) with a forebrain selective reduction of AdoK expression [31], which exhibits a 3.3-fold increase in hippocampal Ado concentration [39], show reduced transmethylation. Intraventricular implantation of Ado-releasing polymers in naive rats reduces global DNA methylation in the hippocampus when compared with that of rats receiving control polymers. The therapeutical administration of Ado in the kainic acid-treated rats results in reversal of the hypermethylation to control levels and in reduction of seizure activity, that is maintained beyond the time window of Ado release. Moreover, inhibition of DNMT reduces seizure susceptibility and epilepsy acquisition, indicating that intracellular Ado concentration, by acting on the transmethylation pathway, plays a role in the modulation of epileptogenesis [38]. Another interesting finding is that increased astroglial AdoK expression in epileptic rats increases 5-methylcytosine immunofluorescence in adjacent neurons. Therefore, the reduction of Ado in astrocytes may affect not only DNA methylation within the astrocytes but also the global Ado, and this, indirectly, could modulate the epigenetic changes in the neighboring neurons [38].

The effect of intracellular Ado through regulation of the transmethylation pathway is not restricted to the nervous system. Another example of the effect of intracellular Ado regulation of the transmethylation pathway in non-nervous tissue is the stimulation of angiogenesis. The involvement of extracellular Ado and Ado receptors in angiogenesis is well established [40,41], but the role of intracellular Ado has recently been highlighted [42]. In vivo studies have demonstrated that hypoxia downregulates AdoK and increases intracellular Ado [43,44]. Xu et al. [42] report that hypoxia increases endothelial intracellular Ado through HIF-1a-dependent AdoK downregulation in vitro and that AdoK knockdown in endothelial cells increases angiogenesis in vitro or ex vivo. Moreover, endothelial AdoK deletion in mice enhances angiogenesis in vivo, as determined by studying the development of retinal vasculature and wound healing response in skin. A2_A_AR and A2_B_AR are the predominant receptors expressed in endothelial cells and have pro-angiogenic effects. Since the combination of A2_A_AR and A2_B_AR antagonists is not able to modify endothelial sprouting and migration, a mechanism independent of these receptors must be responsible for the angiogenic effect. Indeed, AdoK knockdown markedly enhances the levels of intracellular SAH in human endothelial cells, leading to inhibition of transmethylation and accumulation of SAM. Similarly, treatment with Ado causes a significant decrease in DNMT activity in endothelial cells, and the level of 5-methylcytosine is lower in endothelial cells in which AdoK is inhibited than in control cells. The reduction of DNA methylation is not affected by the treatment with A2_A_AR and A2_B_AR antagonists. Therefore, the increase of intracellular Ado might induce hypomethylation of the promoters of angiogenic genes, increasing their expression and promoting angiogenesis. Xu et al. [42] have confirmed this hypothesis. In fact, hypomethylation in the promoter region of the gene coding for the vascular endothelial growth factor receptor 2 (VEGFR2) and other 14 proangiogenic genes has been found in AdoK-deficient human endothelial cells. Accordingly, loss or inactivation of AdoK increases VEGFR2 expression and signaling in endothelial cells, promoting angiogenesis. Although the authors cannot rule out an autocrine or paracrine effect on angiogenesis of extracellular Ado in vivo, the AdoK inhibition-induced high level of intracellular Ado appears to be involved in the stimulation of the endothelial cell growth. In fact, blocking inward transport of Ado using an ENT inhibitor abrogates exogenous Ado-induced VEGFR2 expression [42].

### 3.2. Sleep Homeostasis

Recently, evidence has accumulated indicating that Ado mediates a glial-neuronal circuit that links the glial metabolic state to the A1AR activation of the neurons during homeostatic sleep need. In the adult mice, AdoK is mainly expressed in glia, and the disappearance of neuronal AdoK expression is almost complete by Postnatal Day 14 [45]. When glial Ado increases, it can be transported outside the glial cells through glial ENTs. Then, extracellular Ado activates neuronal Ado receptors, establishing a glial-neuronal circuit regulating the homeostatic sleep buildup and resolution. Homeostatic sleep need refers to the drive to sleep, which increases during waking and decreases after sleeping. Previous waking time is associated with the magnitude of the slow wave activity during sleep that can be measured by electroencephalography. A1ARs are necessary for the increase of slow wave activity after sleep deprivation (rebound). Homeostatic sleep rebound can be reduced by decreasing extracellular Ado concentration either by reducing glial release of ATP, which may be converted to Ado, as reported in transgenic mice expressing a dominant negative SNARE in astrocytes [46] or by knocking out ecto-5′-nucleotidase, the enzyme that converts AMP to Ado [47]. Similarly, the modification of extracellular Ado concentration obtained in transgenic mice overexpressing AdoK in the brain or by inhibiting AdoK results in a decrease and increase of slow wave activity, respectively [48,49]. Knockout of the neuronal A1AR leads to a decreased sleep drive, while the removal of AdoK in neurons does not affect it. Conversely, mice deficient in glial AdoK show an increased homeostatic sleep drive [50,51], as assessed by three sleep indexes (slow wave activity rebound, slow wave decay across and within slow wave activity episodes, and sleep consolidation). Therefore, the Ado-mediated regulation of sleep in response to waking requires activation of neuronal A1ARs, but can be controlled by glial AdoK. Ado appears to link the glial metabolic state and neural-expressed sleep homeostasis.

## 4. Regulatory Mechanisms Mediated by dAdo

dAdo, a natural nucleoside deriving mostly from DNA breakdown and in part from dietary sources, is metabolized by the same enzymes acting on Ado, but is unable to interact with any of the known Ado receptors. This makes dAdo the ideal compound to study the pathways involved in Ado metabolism without taking into account the receptor-mediated effects of the nucleoside. The deoxynucleoside has received major attention because of its central role in SCID. In normal conditions, dAdo is either phosphorylated by AdoK or deaminated by ADA. In the genetic disorder characterized by the absence of ADA, the concentration of dAdo increases and the deoxynucleoside is channeled to the phosphorylation pathway, thus inducing a wide range of toxicities, particularly relevant for the immune system. Indeed, by virtue of high deoxynucleoside-kinase activities and reduced cytosolic 5′-deoxynucleotidase activity, dATP accumulates in lymphocyte [52], and, in turn, exerts a number of toxic effects including inhibition of ribonucleotide reductase [53]. On this basis, inhibitors of ADA are potential immunosuppressive agents. Indeed, deoxycoformycin (dCF), a powerful inhibitor of ADA [54], has found clinical applications for the treatment of several types of lymphatic leukemia [55,56]. Besides leukemia, the combination of dAdo and dCF has been shown to be toxic for several different cell models of tumoral origin [57,58]. In this regard, it has been demonstrated that two human colon carcinoma cell lines (LoVo and HT29) are both sensitive to this drug combination and that the extent of toxicity depends upon the level of enzymes involved both in the process of activation (phosphorylation of dAdo) and inactivation (dephosphorylation of dAMP) of dAdo [59,60]. The combination of dAdo and dCF in LoVo cells causes a block of the cell cycle at the S phase, and cells undergo apoptosis through a pathway involving a dATP-dependent caspase-3 activation [61]. Apoptotic cell death by dAdo and/or Ado has been also reported in sympathetic neurons and adrenal chromaffin cells [62,63], both deriving from the neural crest and sharing several features regarding synthesis, storage, and release of neurohormones [64]. In chick sympathetic neurons, Ado is lethal when added to the culture from the time of plating up to 16 h, and its effect is enhanced by the presence of dCF; thereafter, Ado loses its toxic effect. dAdo instead, both alone and in combination with dCF, is toxic even to several week-old sympathetic neurons. Kulkarni and Wakade [62], except for a distinct transport system for dAdo and Ado, do not find any plausible explanation for this difference, but hypothesize that Ado may have a pro-apoptotic action important in early development. The phosphorylation appears to be an essential step for the two nucleosides to exert their toxic action [62]. Rat chromaffin cells are sensitive to dAdo and dCF in combination, thus confirming the critical role of ADA in the inactivation of a potentially toxic nucleoside. Neither dCF or dAdo alone are toxic, while Ado, both alone or in combination with dCF exerts only a slight insignificant effect. Once again, the phosphorylation of the deoxynucleoside appears to be crucial for its toxic action. Wakade et al. [63] discuss the lack of action of Ado in well-developed rat chromaffin cells, and suggest that specific, but unknown, biochemical pathways leading to apoptotic cell death are modified during development to protect cells from Ado toxicity; in fact, as reported above, Ado kills only freshly plated sympathetic neurons but is ineffective in mature sympathetic neurons [62]. The treatment with dAdo and dCF in combination induces apoptosis in human astrocytoma [65,66] and neuroblastoma cell lines [67], but several differences have been found in the mode of action, possibly reflecting a different functional and metabolic profile of the two cell lines. In astrocytoma cells, an early, still unexplained event preceding the effect of dAdo and dCF on cell viability is a decreased glycolytic capacity, evidenced by a reduction in the production of lactate, while in neuroblastoma cells no effect on glycolytic capacity is observed. An early decrease in mitochondrial ROS production points to an impairment in mitochondrial function in both cell lines, confirmed by an increase in mitochondrial mass, possibly to cope with the decrease in mitochondrial activity. In both cell lines, dAdo must be phosphorylated in order to exert its cytotoxic effect; however, while a profound reduction in ATP level causes a decrease in the energy charge in astrocytoma cells, no apparent effect on energy charge is observed in neuroblastoma cells. Therefore, it is clear that the role of ADA is to avoid the accumulation of the potentially toxic Ado and/or dAdo. In fact, ADA deficiency is a fatal condition and necessitates early intervention, such as allogenic hematopoietic stem cell transplant, enzyme replacement therapy, and hematopoietic stem cell gene therapy. These therapies can correct the abnormalities of the immune system, while the severity of the neurological manifestations persists and appears to increase with age [68]. In this regard, recently, Sauer et al. [69] report a survey performed in untreated ADA-SCID patients as well as in patients after enzyme replacement therapy. Their data confirm that ADA-deficient patients commonly manifest several central nervous system defects such as motor dysfunction, EEG alterations, sensorineural hypoacusia, and low mental development. Enzyme replacement therapy, while partially reducing these manifestations, cannot completely prevent their onset. Sauer et al. [69] extend their study to a model of ADA-deficient mice, which are significantly less active than control littermates and show anxiety-like behavior. Molecular and metabolic analyses indicate that this phenotype shows metabolic alterations, such as an accumulation of Ado, which increases with age, and aberrant receptor signaling, such as hypoexpression of A2_A_AR. Therefore, the authors suggest that the effects of Ado might be mediated by alterations in the arrangement of Ado receptors. Ado concentration in the brain of mice subjected to enzyme replacement is higher than in normal mice, thus indicating that the therapy is unable to lower the nucleoside concentration below the threshold of toxicity for the nervous system. Although not determined, it is conceivable that the concentration of dAdo increases as well, and it is likely that also the deoxynucleoside, through a receptor-independent mechanism, contributes to the onset of the neurological manifestations. In conclusion, although the molecular mechanism underlying Ado and/or dAdo toxicity still remains to be elucidated, these data further confirm the importance of the control over the circulating concentration of the nucleoside.

## 5. Ado as an Energy Source

The brain depends on both glycolysis and mitochondrial oxidative phosphorylation for the maintenance of ATP levels. In fact, several neurodegenerative disorders, such as Alzheimer’s, Huntington’s, and Parkinson’s disease have been related to mitochondrial dysfunction and oxidative stress [70]. Under ATP-depleting conditions, astrocytes and neurons release adenine nucleotides, which are subsequently hydrolyzed to Ado, or Ado itself [71]. Furthermore, in ischemic/hypoxic conditions, the turnover of endogenous nucleotides stemming from dead cells may possibly supply a discrete amount of purine nucleosides [72,73]. Indeed, a number of reports indicates that, following traumatic events, purines are likely to exert trophic effects on neuronal cells through receptor-independent mechanisms. A beneficial effect of purine nucleosides has been observed on the neurite outgrowth of primary rat cerebellar granule cells after hypoxia [74]. Haun et al. [75] have reported that Ado in order to exert its neuroprotective effect on rat astrocyte cultures subjected to combined glucose-oxygen deprivation must be intracellularly converted into inosine. A correlation between protection and ATP preservation has been observed during glucose deprivation and mitochondrial inhibition in rat glial cells treated with purine nucleosides [76]. A similar protective effect of purine nucleosides has been reported by Litsky et al. [77] in primary mixed cultures of murine embryonic spinal cord neurons and glia during chemical hypoxia. In rat primary astrocytes, a receptor-independent protective effect of Ado and purine nucleos(t)ides against the death induced by hydrogen peroxide and glucose deprivation has been also reported [78]. Ado and its deamination product, inosine, like glucose, are able to preserve the ATP pool of human astrocytoma cells previously subjected to mitochondrial inhibition by oligomycin through a receptor-independent mechanism [79].

The protective effect of nucleosides, including Ado, has also been reported in cell types from other tissues, or organs besides brain. It has been demonstrated that, in conditions of energy depletion induced by mitochondrial inhibition, not only do human colon carcinoma cells utilize nucleosides to restore the ATP pool, but their uptake is also increased accordingly with the role of these compounds as an energy source alternative to glucose [80]. In myocardial ischemia, catabolism of Ado appears to mediate the protective effect of pre-conditioning [81]. Finally, in an in vitro model of hepatic ischemia-reperfusion, Módis et al. [82] demonstrate that both Ado and Ino exert cytoprotective effects which are not related to receptor-mediated actions. In fact, they are not prevented by selective Ado receptor antagonists. Moreover, Ado, in order to exert its effect, must be firstly deaminated. 

**Figure 3:**
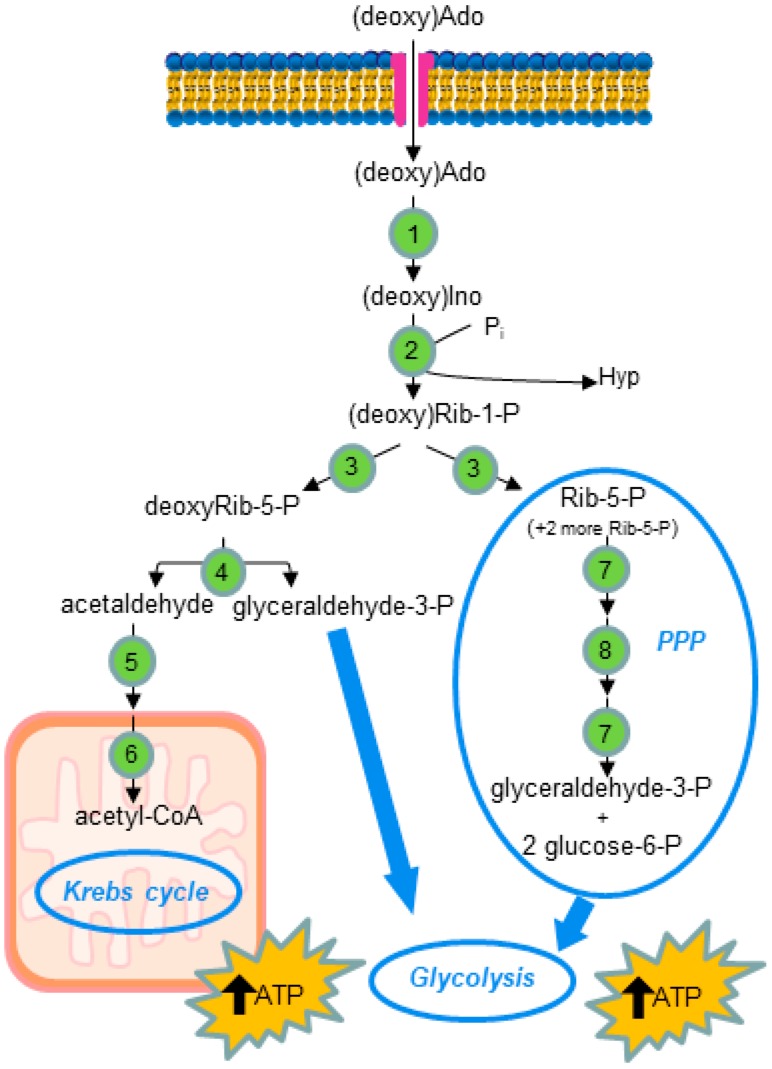
Schematic illustration of the uptake and utilization of (deoxy)Ado. The nucleoside enters the cell through ENT and is subjected to catabolic reactions. The destiny of the ribose moiety of the nucleoside is underlined. 1: Adenosine deaminase; 2: purine nucleoside phosphorylase; 3: phosphoribomutase; 4: deoxyRib-5-P aldolase; 5: aldehyde oxidase; 6: acetyl-CoA synthetase; 7: transketolase; 8: transaldolase. PPP: pentose phosphate pathway (non oxidative branch).

Overall, the literature survey indicates that, although glucose is the major energy source for most cell types, when the glucose supply is lowered and the oxygen is not sufficient to support mitochondrial respiration (ischemic and/or hypoxic conditions), nucleosides may become a major energy source for the cell. Indeed nucleosides can be regarded as carriers of sugar that is made available through the action of PNP; in fact, without energy expense, a phosphorylated compound (Rib-1-P) is generated, which, through the non-oxidative branch of the pentose phosphate pathway and glycolysis, may be converted to energetic intermediates that can be utilized as an energy source (Figure 3).

Since Ado and dAdo are not a substrate of PNP, they are converted by ADA to Ino and deoxyinosine (deoxyIno), respectively, before undergoing the phosphorolytic cleavage of the *N*-glycosidic bond. This step represents the central catabolic reaction [83]. The Rib-1-P formed from Ino can be isomerized into ribose-5-phosphate (Rib-5-P), which can be either utilized for PRPP synthesis or, through the pentose phosphate pathway, can be converted into glycolytic intermediates. Additionally, deoxyIno is subjected to phosphorolytic cleavage of the *N*-glycosidic bond, giving rise to the base and deoxyRib-1-P, which is isomerized to deoxyribose-5-phosphate (deoxyRib-5-P). deoxyRib-5-P may undergo only a catabolic fate, being cleaved by a specific aldolase into acetaldehyde and glyceraldehyde 3-phosphate (glyceraldehyde-3-P) [84]. glyceraldehyde-3-P enters glycolysis, while acetaldehyde may be converted into acetyl-CoA by the action of two enzymes, aldehyde oxidase, and acetyl-CoA synthetase [85]. Therefore, in both cases, the pentose moiety of (deoxy)nucleosides may be utilized for energy repletion (Figure 3).

It is obvious that the metabolic fate of the pentose moiety of nucleosides may help to explain the role of these compounds in preserving different kinds of cells from energy depletion.

## 6. Concluding Remarks

Nucleotides and nucleosides, particularly adenylic compounds, play a number of different regulatory roles spanning from the binding to allosteric sites on enzymes to interaction with specific receptors mediating a complex signaling involved in metabolic regulation, inflammatory mechanisms, proliferation, apoptosis, etc. Distinguishing among the different pathways mediating the effects exerted by the purine compounds is often very complicated. Determining the relative contribution of intracellular and extracellular Ado is currently difficult due to the lack of sufficient spatiotemporal resolution in the techniques available to discriminate between the effects of Ado itself and those of Ado metabolites acting intra- or extracellularly. New tools to interfere selectively and exclusively with a precise pathway are needed. Cellular models and knockout animals for specific adenylate receptors or for enzymes or transport systems have been shown to be very useful in assigning the role played by different proteins in a signaling pathway. However, they also pointed out that a mechanism may be mediated by the interaction of the same signal molecules with different targets, each contributing to the phenomenon at different degrees and through different pathways. Specific enzyme inhibitors or receptor agonists and antagonists have been largely utilized in the studies of purine-mediated cell mechanisms. Usually, these compounds are structurally similar to the natural compounds and as such can interact with many different proteins in an unexpected way, thus leading to wrong conclusions. In this respect, the discrepancies among results obtained in studies using different experimental approaches are not at all surprising. Ado is usually kept at a very low concentration in the body—below 1 µM [2,3]. In a healthy organism, its concentration can increase only as a consequence of ATP breakdown, which can occur both inside or outside the cell. The presence of ENT on the cell membrane ensures a rapid equilibration of the nucleoside concentration. Furthermore, enzymes such as ADA found both inside and outside the cell [86] and AdoK located in the cytoplasm are responsible for the clearance of Ado. This metabolic arrangement endows Ado with the characteristics of a signal molecule that can play its role both interacting with specific receptors but also acting intracellularly on still-unknown molecular targets. Indeed, a role for Ado in the regulation of the transmethylation pathway and in the replenishing of the ATP pool in some critical circumstances has already been revealed and, although with some contradictions, a role of intracellular Ado in the induction of hypothermia has been also reported.
